# Fighting Left Handers Promotes Different Visual Perceptual Strategies than Right Handers: The Study of Eye Movements of Foil Fencers in Attack and Defence

**DOI:** 10.1155/2020/4636271

**Published:** 2020-01-10

**Authors:** Mateusz Witkowski, Ewa Tomczak, Maciej Łuczak, Michał Bronikowski, Maciej Tomczak

**Affiliations:** ^1^Adam Mickiewicz University, Poznań, Poland; ^2^Faculty of English, Adam Mickiewicz University, Poznań, Poland; ^3^Poznań University of Physical Education, Poznań, Poland

## Abstract

Left handers have long held the edge over right handers in one-on-one interactive combat sports. Particularly in fencing, top rankings show a relatively strong overrepresentation of left handers over right handers. Whether this can be attributed to perceptual strategies used by fencers in their bouts remains to be established. This study aims to verify whether right-handed fencers assess their opponents' behaviour based on different perceptual strategies when fencing a left vs. right hander. Twelve top-level (i.e., Olympic fencers, Junior World Team Fencing Champions, and top Polish senior foil fencers) right-handed female foil fencers (aged 16–30 years) took part in the study. They performed a total of 40 actions: 10 repetitions of offensive actions (attack) and 10 repetitions of defensive actions (defence), each type of action performed under 2 conditions (right- vs. left-handed opponent). While the participants were fencing, their eye movements were being recorded with a remote eye-tracker (SMI ETG 2.0). Both in their offensive and defensive actions, the fencers produced more fixations to the armed hand and spent more time observing the armed hand in duels with a left-handed (vs. right-handed) opponent. In defence, it was also the guard that attracted more fixations and gained a longer observation time in bouts with a left hander. In duels with a right-handed opponent, a higher number of fixations in attack and in defence, and longer observation times in defence were found for the upper torso. The results may point to different perceptual strategies employed in bouts with left- vs. right-handed individuals. The findings from this study may help to promote the implementation of specialized perceptual training programmes in foil fencing.

## 1. Introduction

Perception is the fundamental process of gathering information from the external environment [1, 2]. Involved in visual perception, the primary visual system comprises a well-defined area in the human brain along with the surrounding neural pathways [3]. Particularly in sports where the ability to adapt tactical and technical actions to altering conditions determines the final result, the sense of sight is estimated to provide athletes with c. 80% of the information they receive [4]. As a matter of fact, perceptual abilities are reported to be one of the main ability types crucial in sports performance, alongside cognitive and motor abilities. The function of perceptual abilities is to obtain information from the environment and to interpret it in a subsequent action [5]. The way of obtaining information from the environment appears to influence the effectiveness of technical and tactical actions in fencing. Notably, an appropriate perception strategy allows athletes to properly select the distance and anticipate actions of their opponent [6].

In fencing, a sport where time allowed for preparation of motor reactions is very short, top-level fencers anticipate the intentions of their opponents [7]. Such enhanced visual anticipation was found in tennis [8] and squash [9], where expert players extracted information from the very early part of their opponents' actions, particularly arm actions. Here, in visual anticipation of stroke direction, the most critical time periods distinguishing experts from novices were those between 160 and 180 ms before racket-ball contact and the time of extender ball flight occurring at least 80 ms after the contact. Further, investigating anticipation of opponents' bodily movements among badminton players, Wright et al. [10] found expert-novice differences in cortical mechanisms underlying the task of analyzing body kinematics to predict the aim of a badminton shot. Stronger activation in expert players than novices was found in a bilateral set of brain areas associated with all-task-sensitive areas. In novices, on the other hand, voxel clusters with a higher activation on this task turned out to lie outside these areas. Since the regions that responded differentially were early visual areas, kinematic information pick-up appears to be essential for sports-related anticipation.

Along similar lines, in fencing, gaining more time to prepare actions and react adequately is directly linked to early recognition of one's opponent's intentions [11–14]. The ability to early recognize the opponent's actions may—to a greater extent than reaction time alone—differentiate fencers at different levels of sports performance [15]. In foil fencing, an action (i.e., a part of a fencing bout from the referee's command for fencers to begin the fight until a valid hit is scored) takes an average of 5 seconds [16]. Irrespective of the weapon used, during a bout fencers constantly move back and forth on the piste. They react quickly to changing tactical situations and perform a variety of specialized footwork combined with body movements: lunge—a forward movement executed by simultaneously thrusting the front leg forward and pushing off the supporting leg; glide—a forward step ending in a lunge executed with forward acceleration; and fleche—a forward movement executed by crossing the rear leg over the front leg with a simultaneous forward movement of the body (see [17, 18]). Therefore, the way of obtaining information from the surroundings (i.e., kinematic information coming primarily from the opponent) may have a crucial influence on the effectiveness of the undertaken technical and tactical actions. Likewise, an appropriate perceptual strategy can also facilitate imposing one's own combat conditions on the opponent [6]. Indeed, top-level athletes were found to reduce their sensorimotor response time, primarily in the identification and selection of an appropriate response, which translates into improved efficiency of executing specific technical actions in the motor stage [19, 20]. To perform actions, athletes need to constantly adapt to changes in visual information obtained from the environment, including their opponents.

As visual control appears to be fundamental in the process of executing movements [21], the study of visual information processing and gaze behaviour should form an integral part of technical and tactical preparation in fencing. Recording eye movements allows researchers to gain insight into where and for how long an athlete's attention is directed. Identified areas of interest (meaningful regions in visual stimuli) and registered measures underlying visual attentional processes, including fixations—positioning of gaze on a given location for a period of around 200–300 ms, and saccades—very fast eye movements, from 30 to 80 ms, between two successive fixations [22, 23], serve as crucial indicators of how information is obtained during a duel. Such fine-grained measures are informative about visual perceptual strategies (an individual's guided gaze behaviour during the task) employed by fencers in their bouts.

Numerous studies conducted to date have explored gaze behaviour differences related to sports expertise. Empirical evidence shows that during sports activity expert athletes exhibit systematically different visual search behaviours from novices (see [1]). Last three decades have witnessed a great deal of research on expertise-based gaze behaviour that systematically indicates that experts produce fewer fixations of longer duration compared to novices (see [24]). A comprehensive metaanalysis of empirical work on perceptual differences relative to sports expertise [25] shows that apart from short fixations in general, experts also exhibit a greater number of fixations on task-relevant regions and fewer fixations on task-irrelevant regions. In addition, as a result of superior parafoveal processing as well as selective attention allocation, experts' saccades are longer and times to first fixation to task-relevant information are shorter. The presence of systematic expert-novice differences in gaze behaviour has been corroborated by the accumulated research in individual disciplines such as racquet sports [1, 9, 26–28] and shooting sports [29, 30], as well as in team sports [21, 31–35]. Inasmuch as the empirical evidence is robust, only a handful of studies to date have investigated gaze behaviour of fencers in bouts, particularly with an opponent's handedness as a variable.

One of the studies of Hagemann et al. [36], in the laboratory experiment, examined eye movements of 15 expert fencers (top-ranked), 15 advanced fencers, and 32 sport students. The participants' task was to watch fencing attacks on a computer screen and—at the end of each video—to click the anticipated target area. Their viewing time, fixation duration, and number of fixations were recorded for each video-based attack and for each target region. Hagemann et al.'s [36] results showed that expert fencers fixated specifically on the upper trunk (upper torso). However, when the opponent's upper trunk was covered, the fencers also looked at adjacent body regions. Visual perceptual strategies of fencers differed significantly relative to their level of fencing proficiency, which may indicate that the way of obtaining visual information from the environment may have a crucial role to play in their sports achievements. Comparable studies conducted in épée fencing found that professional (expert) fencers exhibited shorter fixation durations in the investigated target areas than novices [37].

Previous reports point to the importance of handedness in interactive one-on-one sports. Loffing and Hagemann's [38] comprehensive summary including the prevalence of left handedness at the elite-level across various sports shows an overrepresentation of left handers in interactive sports (i.e., sports where the action of one athlete directly affects the action of the other), both individual (e.g., fencing, table tennis, judo) and team sports (e.g., cricket, baseball). By contrast, no comparable frequency effects of left-handedness are observed in noninteractive sports (e.g., bowling, darts, golf). Yet another analysis of the ranking data of athletes competing in interactive ball games [39] reveals a clear overrepresentation of left handers (relative to the general human population) at the top-ranked level in baseball, cricket, and table tennis, all being highly time-constrained sports. Interestingly, the same analysis shows no similar prevalence of left-handedness in lower time pressure sports (tennis and squash). Being direct, close, interactive and unilateral, fencing is a sport in which two athletes combat against each other holding a weapon in one arm [40]. In fact, the number of left-handed fencing finalists and championship medalists is relatively high compared to the number of the fencing population and left handers in the general human population. The predominance of left-handed fencers also shows in more recent rankings of the International Fencing Federation (FIE) issued for senior female foil fencers. The analysis of the FIE ranking released at the end of 2016/2017 found the percentage of left-handed female foil fencers in the respective ranking ranges (1–32, 33–64, 65–96, 97–128, 129–160, 161–192, 193–224, 225–256) to be clearly increasing, with 13% for rankings 225–256 and 31% for the top 32 foil fencers [41].

Bearing in mind that left-lateralisation (from moderate to strong) occurs in about 10% of the human population [42, 43], such an overrepresentation of left handers in top rankings is thought-provoking. Laterality researchers fairly consistently credit the left handers' advantage to their smaller number in the fencing population, or in other words, to the frequency-dependency effects (see [44] for an overview of existing theories on left-handed fencing). That left-handed fencers, comprising the minority in the fencing population, are used to confronting right-handed competitors reflects in their more effective combat strategies [45], compared to right handers who have fewer opportunities to fence left handers. It remains to be established, however, whether the reasons behind that advantage can specifically be linked to visual-motor control mechanisms and perceptual strategies employed by right handers in bouts with left-handed opponents.

In fencing, there are two main categories of actions: preparatory and actual. Preparatory actions include postures, movements, and tactical fake attacks intended to mislead the opponent. Recognizing the opponent's intentions and abilities, concealing one's own intentions and deceiving the opponent, hindering the opponent's concentration, impeding the execution of their actions, keeping the right distance to attack or defend, facilitating the execution of actual actions all fall into the scope of preparatory actions. The actual fencing actions are classified into offensive, defensive, and counter-offensive. Offensive actions comprise attacks, ripostes, counterripostes, and varieties of relaunched offensive actions. Defensive actions, on the other hand, include parries, defence distance, and evasions. Counter-offensive actions include lines and counterattacks [20]. Executing the actual actions in defence and in attack involves the orientation of the perceptual apparatus and immediately precedes the stage where the action is completed and a point is scored (or lost). Defensive actions tend to be more reactive. The quality of the reaction to the opponent's actions in a reactive defensive situation (preceded by stimulus reception and processing) appears to depend on the prior orientation of fencer's perception and feeds into accuracy and speed of reaction(s) during actions exchanged at very close distances. Here, orientation of a fencer's perception may differ relative to an opponent's handedness. Offensive actions, on the other hand, tend to be more proactive. Here, an attacker's focus and perception, oriented outwards, contribute to the identification of the target area. Given that left handers' body postures and weapon positions differ from right handers', the attacker's perception may also orient differently. Altogether, owing to these differences, fencers may perceive bouts with left-handed opponents as less familiar and more complex. The level of task complexity (e.g., viewing task, detection task, decision task, or problem-solving task) and task novelty, in turn, have been reported to influence athletes' visual searches (for further discussion of differences in eye movements varying as a function of task complexity see [25]).

From the body of empirical work conducted over the past three decades emerges a relatively clear picture that attention is guided by scene properties (bottom-up, stimulus-driven guidance), the known feature (top-down, user-driven guidance), scene structure and meaning, history of prior search (prior experience of the observer), and the perceived value (or reward) of the targets and distractors (for an overview see [46]). A recent systematic review of theories relating to the interplay of expert visual search behaviour and their perceptual-cognitive skills points to three major theories that help to explain how the processes underlying perceptual-cognitive skills and expertise may affect expert performance (see [47]): (1) the long-term working memory theory, (2) the information-reduction hypothesis, and (3) the holistic model of image perception. Following these theoretical accounts, compared to less-experienced individuals, expert athletes in fencing can be assumed to (1) encode and retrieve visual information from their long-term memory more efficiently, (2) optimise the amount of information that they process by selectively allocating their attention to task-relevant cues, concurrently ignoring task-irrelevant stimuli, and (3) enjoy superior global-local image processing in that they obtain more visual information from parafoveal and distal areas and thus show more efficient holistic processing of the scene.

Given that, duels with a rarer and less familiar type of opponent (i.e., left hander) may also keep athletes' anxiety levels high. In line with Attentional Control Theory (ACT) [48], through a disruption of the balance between the two fundamental attentional systems, anxiety impairs both processing efficiency and efficiency of attentional control. The ACT assumes that the presence of a threatening stimulus decreases goal-oriented (top-down) attention and, at the same time, increases stimulus-driven (bottom-up) attention. Since anxiety alters the balance of these two attentional systems, it affects processing efficiency and visual search behaviour. More complex, more novel, and anxiety-inducing tasks promote gaze behaviour oriented towards peripheral body regions (e.g., limbs), whereas simpler tasks elicit looking patters that are directed towards central body areas (e.g., chest). Similar differences in perceptual strategies in duels with left- vs. right-handed opponents were reported for preparatory actions in foil fencing. In bouts with left-handed opponents, right-handed fencers produced a higher number of fixations to their opponent's peripheral body region (armed hand) and spent more time watching it. Conversely, in bouts with right-handed opponents it was the upper torso (i.e., central body region) that attracted more fixations and longer observation times [49].

The present study aims at describing and comparing visual perceptual strategies in a group of top-level Polish foil fencers (right-handed) during bouts (attack and defence) with right- and left-handed opponents. To achieve this goal, we used a remote eye-tracker to record the participants' eye movements during their fencing actions. On the whole, an eye-tracker, among various eye movements, registers fixations (the state when the eye remains relatively still in a position over a period of time) and saccades (the rapid motion of the eye from one fixation to another) (see [23]). Owing to its high ecological validity, eye-tracking technologies allowing unrestricted head movements appear to be a very valuable tool in research on athletes' gaze behaviour in real-life sports situations (here, fencing bouts). Indeed, eye movement studies capitalizing on remote eye-tracking allow unrestrained movements during fencing bouts in naturalistic settings, and, by the same token, allow very little (or no) researcher interference in fencers' naturalistic interactions on the piste. Along with the representativeness of the investigated fencing actions, these factors improve the prospects of generalizability and application of our research results.

It was hypothesized that right-handed fencers would differently orient their attention and assess the situational factors when fencing a left-handed than right-handed opponent. To verify the hypothesis, an experimental study design was employed.

## 2. Materials and Methods

### 2.1. Participants

A group of 12 top-level female foil fencers aged 16–30 years (*M* = 20.86; SD = 4.76) participated in the study. All the participants (*n* = 12) had normal or corrected to normal vision, and self-declared right-handedness for fencing bouts and training. Additionally, the Edinburgh Handedness Inventory [50] confirmed their right hand dominance in everyday activities. The participants (top-level foil fencers) consisted, among others, in Olympic fencers, Junior World Team Fencing Champions, and top Polish senior foil fencers, all with high-level fencing skills and comparable experience as fencers. The participants gave their written informed consent to participate in the study. The research was approved by the Bioethics Committee of Poznań University of Medical Sciences in Poland (reference no. 982/17).

### 2.2. Apparatus

The fencers' eye movements were recorded with a remote SensoMotoric Instruments Eye Tracking Glasses 2.0 (SMI—ETG 2.0), an eye-tracker with a sampling rate of 60 Hz, a native resolution of 1280 × 960 pixels, and automatic parallax compensation. For data management and analysis, we used the SMI BeGaze™ analysis software ver. 3.7. The choice of an eye-tracker was guided by the need to collect data in a setting mirroring the conditions of real-life fencing bouts (i.e., duels on the fencing piste), thereby striving for relatively high ecological validity of the study.

### 2.3. Study Procedures

Prior to the study, all the participants were informed and instructed on the study procedure. Upon fitting the eye-tracking glasses on their heads, a three-point calibration was performed for each. The study was conducted on the fencing piste in a well-lit sports facility. For each participant, the study involved 2 tasks with predetermined fencing actions: offensive actions (attack) and defensive actions (defence), and 2 conditions: a bout with a right-handed opponent and a bout with a left-handed opponent. Two female opponents took part in the study: one right-handed and the other left-handed, both at a highly advanced level in foil fencing. They were comparable in terms of anthropometric features, including body height and weight. Each participant was informed what actions were to be carried out and in which sequence. Each foil fencer performed 10 repetitions of each task, one after another (10 offensive actions and 10 defensive actions), under each condition, which altogether amounted to 40 actions (20 attacks and 20 defences). Each individual action was performed following the referee's command for fencers to begin a bout (*Ready*, *Go*). All attacks ended in a lunge and all defensive actions ended in a simple touch in the target area. The sequence of the conditions (i.e., right- vs. left-handed opponent) was randomized, so was the order of tasks (attack vs. defence). The research was carried out in cooperation with the Institute of Sensory Analysis based in Warsaw.

### 2.4. Reference Image

**Figure 1:**
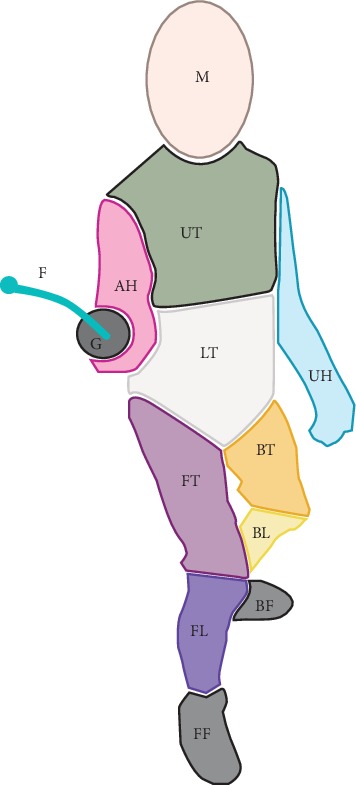
Reference image with areas of interest (AOIs).

In view of the procedure outlined above, six reference images were prepared and utilized, depending on the task (2 images) and study condition (2 images). Each reference image was annotated with areas of interest (AOIs, see Figure 1), following the key presented below:

- *G*—*guard*
- *F*—*foil (blade and tip)*
- *M*—*mask*
- *AH*—*armed hand*
- UH—unarmed hand
- *LT*—*lower torso*
- *UT*—*upper torso*
- *FT*—*front thigh*
- BT—back thigh
- FL—front leg
- BL—back leg
- FF—front foot
- BF—back foot

Based on the preliminary analysis of all the areas, the regions that attracted attention of all participants were identified (a selection criterion: at least 5 glances per AOI produced by a fencer during the whole experiment). The AOIs thus identified (highlighted in italics) were subjected to further analyses.

### 2.5. Statistical Analyses

To verify the hypothesis that fencers differ in the way they look at their opponents relative to their opponent's handedness, a two-way within-subject analysis of variance (ANOVA) was performed on the data, with *AOI* and *opponent's handedness* as the within-subject factors. While the first within-subject factor comprised the following seven levels: *guard* (G), *foil* (*blade and tip*) (F), *mask* (M), *armed hand* (AH), *lower torso* (LT), *upper torso* (UT), and *front thigh* (FT), the second two-level factor related to an opponent's handedness embraced: *left-handed* (LH) and *right-handed* (RH). In the cases where data violated the sphericity assumption, the Greenhouse–Geisser correction was applied. In accordance with the hypothesis, two types of effects were primarily analyzed: interaction effects between the two within-subject factors and main effect of *opponent's handedness*. Significant interaction effects of the two factors indicate that the differences in the mean values of the dependent variables found for the identified AOIs depend on the opponent's handedness. Bonferroni post hoc tests were applied where interaction and main effects were found to be significant. Statistical analyses, performed using IBM SPSS 23, were computed for three dependent variables (eye-tracking metrics):

- *dwell time* (%)—the time devoted to a given AOI expressed in percentage points
- *average fixation*—average fixation duration in an AOI (normalized)
- *fixation count*—the number of individual eye fixations within an AOI (normalized)

In line with the research procedure, the foil fencers were tested under two conditions: in attack and in defence. For each condition and each participant, three eye-tracking metrics were calculated for the identified AOIs relative to their opponent's handedness. Two indicators (*average fixation* and *fixation count*) were normalized by transforming the eye-tracking data into their natural logarithms. The statistical analyses were conducted by the Institute of Sensory Analysis based in Warsaw.

## 3. Study Results

### 3.1. Offensive Actions (Attack)

For *dwell time (%)*, the analyses revealed a statistically significant interaction effect between *AOI* and *opponent's handedness*, *F*(1.66, 18.27) = 4.78, *p* < 0.05, *η*_*p*_^2^ = 0.30 (Greenhouse-Geisser correction, *ε* = 0.27). The foil fencers spent significantly more time looking at their opponent's armed hand when they fenced a left-handed than right-handed opponent (*p* < 0.05) (see Table 1). In duels with a right-handed competitor, however, they looked significantly longer at the upper torso than at most other identified areas of interest, except for the lower torso where the difference did not reach statistical significance. Similarly, in duels with a left-handed opponent, the foil fencers spent significantly more time watching their opponent's upper torso than most other AOIs. However, no statistically significant differences were observed for comparisons made with the armed hand and lower torso (detailed comparisons are presented in Table 1). While the main effect of *opponent's handedness* was not significant, *F*(1, 11) = 0.001, *p* > 0.05, the main effect of *AOI* reached significance, *F*(1.95, 21.49) = 22.4, *p* < 0.001, *η*_*p*_^2^ = 0.67 (Greenhouse–Geisser correction, *ε* = 0.33). The obtained interaction effect, however, renders the main effect of *AOI* less relevant (see Table 1 for detailed comparisons).

For *average fixation*, the performed analyses did not show a significant interaction effect between *AOI* and *opponent's handedness*, *F*(2.71, 29.78) = 1.44, *p* > 0.05 (Greenhouse–Geisser correction, *ε* = 0.45), or a significant main effect of *opponent's handedness*, *F*(1, 11) = 2.61, *p* > 0.05. The main effect of *AOI* was found to be statistically significant, *F*(6, 66) = 14.03, *p* < 0.001, *η*_*p*_^2^=0.56 (for detailed comparisons, see Table 1).

For *fixation count*, a statistically significant interaction effect between *AOI* and *opponent's handedness* was recorded, *F*(6, 66) = 9.95, *p* < 0.001, *η*_*p*_^2^ = 0.48. A significantly higher number of fixations to the opponent's armed hand was observed in duels with the left hander than that of the right hander (*p* < 0.01). In combats against a right-handed competitor, however, a significantly higher number of fixations to the upper torso was registered relative to the left-handed opponent condition (*p* < 0.05). Then, in bouts with a left-handed opponent, the upper torso and the armed hand attracted a significantly higher number of fixations compared to most other areas of interest. Conversely, in confronting the right-handed, significantly more fixations were recorded to the upper torso than to most other areas of interest (for detailed comparisons, see Table 1). The main effect of *opponent's handedness* did not reach statistical significance, *F*(1, 11) = 3.50, *p* > 0.05, whereas the main effect of *AOI* was observed to be significant, *F*(2.38, 26.18) = 31.89, *p* < 0.001, *η*_*p*_^2^ = 0.74 (Greenhouse–Geisser correction, *ε* = 0.40). The latter, however, is believed to be of less importance due to the reported significant interaction effect (more detailed comparisons are shown in Table 1).

### 3.2. Defensive Actions (Defence)

For *dwell time* (%), a statistically significant interaction effect between *AOI* and *opponent's handedness* was found, *F*(1.50, 16.46) = 11.66, *p* < 0.01, *η*_*p*_^2^ = 0.52 (Greenhouse–Geisser correction, *ε* = 0.25). The foil fencers spent significantly more time looking at their opponent's armed hand (*p* < 0.01) and guard (*p* < 0.05) when their competitor was left-handed rather than right-handed. In duels with the right-handed, however, they looked for a longer time at the upper torso (*p* < 0.01) and the lower torso (*p* < 0.05) than that when competing the left-handed. While fencing a left-handed opponent, the foil fencers spent significantly more time watching the armed hand compared to most other areas of interest. During combats against the right-handed, they looked at the upper torso for a significantly longer time than at most other identified areas (see Table 2). The analyses did not show the main effect of *opponent's handedness* to be statistically significant, *F*(1, 11) = 0.71, *p* > 0.05. However, they revealed a significant main effect of *AOI*, *F*(2.14, 23.50) = 16.48, *p* < 0.001, *η*_*p*_^2^ = 0.60 (Greenhouse–Geisser correction, *ε* = 0.36), which is of less relevance given the significant effect of interaction (see Table 2 for detailed comparisons).

For *average fixation*, the interaction effect between *AOI* and *opponent's handedness* did not reach statistical significance, *F*(2.85, 31.33) = 1.85, *p* > 0.05 (Greenhouse–Geisser correction, *ε* = 0.48). The main effect of *opponent's handedness* was not observed to be statistically significant as well, *F*(1, 11) = 2.65, *p* > 0.05, which indicates no statistically significant differences in average fixation duration depending on the opponent's handedness in general, or the opponent's handedness by the identified AOI. However, the main effect of *AOI* was statistically significant, *F*(2.73, 30.05) = 28.57, *p* < 0.001, *η*_*p*_^2^ = 0.72 (Greenhouse–Geisser correction, *ε* = 0.46). See Table 2 for detailed comparisons.

For *fixation count*, the performed analyses showed the interaction effect between *AOI* and *opponent's handedness* to be statistically significant, *F*(2.74, 30.10) = 9.39, *p* < 0.001, *η*_*p*_^2^ = 0.46 (Greenhouse–Geisser correction, *ε* = 0.46). In duels with the left-handed, significantly more fixations were recorded in the area of the opponent's guard (*p* < 0.01) and armed hand (*p* < 0.001) than in duels with the right-handed. A higher number of fixations to the opponent's upper torso, however, was observed during duels with a right-handed competitor (*p* < 0.01). In addition, in bouts with the left-handed, significantly more fixations to the opponent's armed hand and guard were recorded than to most other analyzed AOIs. On the other hand, when fencing the right-handed, significantly more fixations were registered to the opponent's upper torso and armed hand, compared to the majority of other identified areas of interest (more detailed comparisons are shown in Table 2). The main effect of *opponent's handedness* was significant, *F*(1, 11) = 5.79, *p* < 0.05, *η*_*p*_^2^ = 0.35. A higher number of fixations was recorded during duels with a left-handed opponent. In addition, a statistically significant main effect of *AOI* was obtained, *F*(2.51, 27.58) = 33.70, *p* < 0.001, *η*_*p*_^2^ = 0.75 (Greenhouse–Geisser correction, *ε* = 0.42). Nevertheless, the main effects revealed in the analyses are less meaningful due to the significant interaction effect between *AOI* and *opponent's handedness*. For more detailed comparisons, see Table 2.

## 4. Discussion

In the present study investigating the top-level Polish foil fencers in bouts, we found differences in visual perceptual strategies relative to an opponent's handedness. In offensive actions, it was the armed hand where the main differences between bouts with left- and right-handed opponents were observed. More specifically, when duelling left-handed opponents, the foil fencers spent more time observing the armed hand and produced a higher number of fixations to the armed hand relative to the right-handed opponent condition. Additionally, in duels with left-handed opponents, the armed hand attracted more fixations than most other areas of interest identified in the study. Relatively comparable results were obtained for defensive actions, where in bouts with left-handed opponents, again, we recorded more fixations as well as longer observation times to the armed hand and, additionally, to the guard compared to bouts with right-handed opponents. Conversely, in combats against right-handed competitors, the upper torso attracted a higher number of fixations in attack and more fixations and longer observation times in defence compared to the left-handed opponent condition. Only in the case of average fixation duration, however, no significant differences were found relative to an opponent's handedness or performed task type (i.e., offensive vs. defensive actions). The differences reported here were limited solely to the identified areas of interest.

The obtained results could be explained by looking at how different task types yield different perceptual strategies. Previous research shows that perceptual strategies of expert and novice athletes depend on a level of task complexity [24, 25] and novelty [51]. Since duels with left-handed opponents are considerably less frequent, we can assume that fencing a left hander represents a more complex task than fencing a right hander. Hence, bouts with a left-handed opponent yield more fixations to the armed hand than to the upper torso.

Such an interpretation of our results remains fairly consistent with the assumptions of the Attentional Control Theory [48]. The theory proposes that anxiety leads to an increased influence of the stimulus-driven attentional system (bottom-up, peripheral) over the goal-oriented (top-down, central) attentional system. Anxiety is also perceived as enhancing the level of attention given to threat-related stimuli. In line with these assumptions, fencing a left-handed opponent, who is less frequent and thus viewed as less predictable in their actions, may evoke a higher level of anxiety. This results in a shift in visual search strategy from central (e.g., upper torso) to peripheral body areas (e.g., armed hand), recorded as increased concentration on the stimuli regarded as threatening. Interestingly, a landmark study by Williams and Elliott [51] found that increased anxiety of karate performers affected their visual search strategies significantly: they directed their visual attention from central (e.g., chest) to more peripheral body regions (e.g., limbs).

Generally, the results of the study clearly support the hypothesis that different visual perceptual strategies employed during fencing bouts depend on an opponent's handedness. The study by Hagemann et al. [36] into visual perceptual strategies of three groups of fencers of different expertise found that in duels with a computer-generated right-handed opponent, expert fencers primarily fixated on the upper torso and when the upper torso was covered, they looked at the adjacent body regions. The results of our study, however, imply that whether the top-level (expert) fencers focus on the upper torso depends on their opponent's handedness.

In view of the fact that left-handed fencers are considerably less numerous than the right-handed in the fencing population, it can be assumed that fighting conditions peculiar to bouts with left handers are atypical. A left-handed fencer, with their armed hand and weapon positioned on the opposite side, initiates and executes actions on the other side compared to a right-handed fencer. As a result, a left hander's opponent, prepared that in bouts with a right-handed fencer, for instance, a hit on the torso will be initiated on the left side, has to shift their perception and attention. Also, most actions that follow from the fencer's blade entail a change of perception. A similar pattern holds for actions performed in attack, where left-handed opponents execute defensive actions on the opposite side rather than the side their attackers are used to. Changing one's perception, in turn, may entail additional costs including slower reaction times or, in a bout requiring complex actions, less effective management of habits. Reduced effectiveness may be related here to involuntary activation of automatisms typical of bouts with right-handed competitors, yet inadequate in duels with left-handed opponents. Therefore, due to the fact that bouts with left-handed fencers are less frequent, thus more unusual and involving more difficult fighting conditions, fencers may develop other perceptual strategies, primarily those related to the perceptual control of threat that, in this case, is detected first owing to the movement of the armed hand or the guard. These visual perceptual strategies employed in duels with the left-handed may as well be related to fencers' less effective looking patters. Interestingly, one of the main differences between offensive and defensive actual actions in duels with the left-handed is that foil fencers pay more attention to the guard in defence than in attack. As indicated in the introduction, this could be attributed to the more reactive nature of defensive than offensive actions. Earlier control of the guard area may allow fencers to pick-up signals from their left-handed opponents' weapon faster and more accurately.

Several limitations of the present study should be acknowledged. Since the aim of our study is centred on the relationship between an opponent's handedness and employed perceptual strategies, the quality of the participants' actions and their motor performance (including their success rate) was not taken into account in data analyses. Pairing specific actions with their success rate would help to render a more comprehensive picture of how visual perceptual strategies may relate to the effectiveness of the undertaken actions relative to an opponent's handedness. This may be viewed as one of the limitations of our study. Also, a further analysis of eye-tracking data with regard to key movements in offensive and defensive actual actions (e.g., the onset of an individual defensive action) would shed more light on how the direction and timing of top-level fencers' visual attention might be affected by their opponents' handedness. To afford a yet fuller picture of gaze behaviour in fencers under various fighting conditions, future studies may also wish to explore and discuss additional eye-tracking metrics such as *search rate* (i.e., number of fixations and fixation duration irrespective of AOIs) or *time to first fixation* on AOI (see [47]).

The current study investigated the nature of visual searches solely among top-level foil fencers (experts). Our findings and conclusions can thus be applied to a specific group of athletes: high-level performance female foil fencers. That could be viewed as a potential limitation of the current study. We investigated top-ranked foil fencers, whose number is very limited. Given the obtained effect sizes, we believe that despite the small sample size in our study, our findings pointing to notable differences in perceptual strategies in bouts with a left-handed and right-handed opponent allow for practical considerations for training experienced foil fencers and for formulating new hypotheses. Future research of fencers' perceptual strategies would benefit from incorporating a reference group of novices and a group of intermediate-level less-experienced foil fencers into the study design. Not only would it widen the generalizability of the findings to a larger population of foil fencers, but it would also enable researchers to precisely address the questions of how the level of sports expertise modulates visual search strategies (perceptual expertise). Finding that anxiety has a more profound negative impact on less-experienced athletes would substantiate the assumption that it is anxiety that impairs visual attention and processing in fencers. In a similar vein, future empirical work on foil fencers could examine the interplay of ranking positions and visual search behaviour. Building upon the existing work and refining the study design further, future research could recruit left-handed alongside right-handed fencers as participants. Each group of participants (right vs. left handers) would then be assigned to two fighting conditions (fencing right- vs. left-handed opponents).

Modern fencing comprises three subdisciplines (foil, épée, and sabre). They differ substantially from one another, having different target areas and following different rules for awarding points when both fencers perform simultaneous hits on their opponent. This has implications both for refereeing individual fencing bouts and for training fencers to achieve high performance. The present study focused on foil fencers alone; therefore, a fruitful direction for further research is to develop a better understanding of visual search strategies employed by épée and sabre fencers.

In our study, we recorded and analyzed eye movement data, which may make the interpretation of the obtained data challenging. For this reason, several issues need to be addressed. First, while directing one's eyes towards a specific area, information from neighbouring areas can also be retrieved [52]. Second, the range of information received through the central retina of the eye, the belt surrounding the fovea, and the peripheral regions of the retina [53] has not yet been very clearly defined. Williams and Ericsson [54] highlight that the parafovea and visual periphery are used to extract information from regions neighbouring the fixated area, and thus eye movement data need to be interpreted with caution. Third, while experiments using remote head-unrestrained eye-trackers enjoy high ecological validity, they have also been reported to have their shortcomings. Indeed, compared to experimental settings where head-mounted eye-trackers and chinrests are used, tracking participants' gaze behaviour in less optimal conditions (i.e., when participants' heads are unrestrained) may result in data quality deterioration, especially in terms of the amount of data loss and the accuracy of the registered gaze position (for comparison of performance of several remote eye-trackers see [55]).

Visual perception plays a crucial role in competition and training in most sport disciplines. In fencing, duelling fencers watch each other's actions and adapt their own technical and tactical actions to their opponent's. A comparable situation occurs during training, in particular during individual practice with the coach. Findings from our study thus yield practical implications for individual training sessions in fencing honing technical and tactical elements that are then used in real-life fencing matches. The coach lays out various action scenarios, and the fencer attempts to find the best ways to correctly deliver a scoring hit. During such sessions, the coach fences using their right as well as left hand to provide adequate conditions and to make the training session as realistic as possible. Selecting an appropriate strategy, in fact, depends, among others, on an opponent's handedness. In this way, research on visual perceptual strategies among fencers may form the basis that would motivate planning of combat strategies and individual training sessions with right- and left-handed fencers. Knowledge about the sources of visual information in a fencing bout is also essential to the teaching context where fencing techniques are taught to right- and left-handed fencers. Enhancing visual perception of the best athletes has thus the potential to be the key factor giving them the edge over their competitors.

## 5. Conclusions

The study points to the primary differences in visual perception of fencers relative to their opponent's handedness. Both in attack and defence in bouts with left-handed competitors, female foil fencers made more fixations to the armed hand and spent more time observing the armed hand in general than in duels with the right-handed. In defensive actions, it was also the guard that attracted more fixations and gained longer observation times in duels with left-handed versus right-handed opponents. In bouts with the right-handed, on the other hand, the upper torso drew longer observation times and a higher number of fixations in defence as well as more fixations in attack relative to duels with the left-handed. The obtained results may point to different perceptual strategies employed by top-level foil fencers in bouts with left-handed compared to right-handed opponents.

## Figures and Tables

**Table 1:** Comparisons of the means calculated for the eye metrics (*dwell time %*, *average fixation*, and *fixation count*) for the identified AOIs relative to the opponent's handedness—*offensive actions*.

Hand	AOI							
	G	F	M	AH	LT	UT	FT	Total
*Dwell time (%)*								
LH	3.94_b_ (5.63)^#^	1.14_c_ (2.64)	0.16_d_ (0.25)	22.40_a_ (24.39)	8.69 (17.49)	39.18_b,c,d,e_ (26.55)	0.54_e_ (1.75)	10.87 (20.17)
RH	2.99_f_ (6.85)	0.29_g_ (0.46)	1.23_h_ (2.10)	3.14_a,i_ (6.81)	12.28 (17.60)	56.13_f,g,h,i,j_ (27.49)	0.03_j_ (0.12)	10.87 (22.69)
Total	3.47_4_ (6.15)	0.72_3_ (1.90)	0.70_2_ (1.56)	12.77_5_ (20.09)	10.49 (17.25)	47.65_1,2,3,4,5_ (27.81)	0.29_1_ (1.24)	— —

*Average fixation*								
LH	3.89 (2.90)	1.83 (2.71)	1.69 (2.51)	5.68 (0.71)	4.59 (2.18)	5.65 (0.51)	0.94 (2.21)	3.47 (2.77)
RH	2.79 (2.93)	1.86 (2.75)	1.86 (2.75)	3.47 (2.62)	4.74 (2.28)	5.81 (0.33)	0.47 (1.61)	3.00 (2.83)
Total	3.34_9_ (2.90)	1.84_3,8_ (2.67)	1.77_2,5,7_ (2.57)	4.58_6,7,8_ (2.19)	4.66_4,5_ (2.19)	5.73_1,2,3_ (0.43)	0.70_1,4,6,9_ (1.91)	— —

*Fixation count*								
LH	0.93_c,d_ (0.98)	0.32_e,f_ (0.79)	0.00_g,h_ (0.00)	2.57_a,c,e,g,i_ (0.94)	1.31_j_ (1.28)	3.30_b,d,f,h,j,k_ (0.88)	0.12 _i,k_ (0.40)	1.22 (1.44)
RH	0.69_l_ (1.03)	0.06_m,p_ (0.20)	0.49_n_ (0.82)	0.75_a,o_ (1.01)	1.81_p,r_ (1.43)	3.84 _b,l,m,n,o,s_ (0.55)	0.00_r,s_ (0.00)	1.09 (1.50)
Total	0.82_4_ (0.17)	0.19_3,9_ (0.19)	0.25_2,8_ (0.25)	1.66_5,7,8,9_ (1.29)	1.56_6,10_ (0.35)	3.57_1,2,3,4,5,6_ (0.38)	0.06_1,7,10_ (0.08)	— —

AOI—area of interest; G—guard; F—foil (blade and tip); M—mask; AH—armed hand; LT—lower torso; UT—upper torso; FT—front thigh; LH—left-handed; RH—right-handed. ^#^Standard deviation is given in parentheses. Subscript letters stand for the statistically significant differences for significant interaction effects AOI *x* opponent's handedness. Subscript numbers stand for the statistically significant differences for significant main effects of AOI. For *dwell time (%)*, ^*∗*^*p* < 0.05—a,b,5, ^*∗∗*^*p* < 0.01—c,d,e, ^*∗∗∗*^*p* < 0.001—f,g,h,i,j,1,2,3,4. For *average fixation*: ^*∗*^*p* < 0.05—5,7,8,9, ^*∗∗*^*p* < 0.01—2,3,4,6, ^*∗∗∗*^*p* < 0.001—1. For *fixation count*, ^*∗*^*p* < 0.05—b,j,p,r,6,10, ^*∗∗*^*p* < 0.01—a,c,d,5,8, ^*∗∗∗*^*p* < 0.001—e,f,g,h,i,k,l,m,n,o,s,1,2,3,4,7,9.

**Table 2:** Comparisons of the means calculated for the eye metrics (*dwell time %*, *average fixation*, and *fixation count*) for the identified AOIs relative to the opponent's handedness—*defensive actions*.

Hand	AOI							
	G	F	M	AH	LT	UT	FT	Total
*Dwell time (%)*								
LH	16.33_a,e,f,g_ (13.15)^#^	3.93_h_ (5.35)	0.54_e,i_ (1.27)	38.82_b,h,i,j,k_ (22.31)	5.00_c,f,j_ (6.46)	20.90_d_ (22.35)	0.18_g,k_ (0.49)	12.24 (18.40)
RH	9.03_a_ (14.96)	1.48_l_ (3.56)	0.00_m_ (0.00)	9.56_b_ (14.91)	13.12_c,n_ (12.53)	49.88_d,l,m,n,o_ (26.64)	0.26_o_ (0.89)	11.90 (21.03)
Total	12.68 (14.27)	2.70_3,7_ (4.62)	0.27_2,6_ (0.92)	24.19_5,6,7_ (23.82)	9.06_4,8_ (10.59)	35.39_1,2,3,4_ (28.24)	0.22_1,5,8_ (0.71)	— —

*Average fixation*								
LH	6.10 (0.49)	4.16 (3.23)	0.89 (2.08)	5.98 (0.54)	4.79 (2.31)	4.88 (2.32)	0.95 (2.23)	3.96 (2.87)
RH	5.35 (1.74)	2.36 (2.95)	0.00 (0.00)	5.76 (0.52)	5.40 (1.87)	5.99 (0.42)	0.58 (2.00)	3.63 (2.90)
Total	5.72_2,6_ (1.31)	3.26 (3.16)	0.45_1,2,3,4_ (1.51)	5.87_1,5_ (0.53)	5.09_4,7_ (2.08)	5.44_3,8_ (1.73)	0.76_5,6,7,8_ (2.08)	— —

*Fixation count*								
LH	2.17_a,d,e,f,g_ (1.00)	0.50_d,h_ (0.80)	0.26_e,i,m_ (0.62)	3.28_b,f,h,i,j,k_ (0.63)	1.14_j,l_ (0.94)	2.34_c,m,n_ (1.42)	0.00_g,k,l,n_ (0.00)	1.39 (1.42)
RH	1.26_a,o_ (1.22)	0.31_p,r_ (0.68)	0.00_s,t,u_ (0.00)	1.73_b,p,s,w,x_ (0.82)	1.77_t,y_ (1.38)	3.58_c,o,r,u,w,z_ (0.78)	0.00_x,y,z_ (0.00)	1.23 (1.45)
Total	1.72_7,8,9_ (1.19)	0.41_3,6,9_ (0.73)	0.13_2,5,8,11_ (0.45)	2.51_4,5,6_ (1.07)	1.45_10,11_ (1.20)	2.96_1,2,3_ (1.29)	0.00_1,4,7,10_ (0.00)	— —

AOI—area of interest; G—guard; F—foil (blade and tip); M—mask; AH—armed hand; LT—lower torso; UT—upper torso; FT—front thigh; LH—left-handed; RH—right-handed. ^#^Standard deviation is given in parentheses. Subscript letters stand for the statistically significant differences for significant interaction effects AOI *x* opponent's handedness. Subscript numbers stand for the statistically significant differences for significant main effects of AOI. For *dwell time (%)*, ^*∗*^*p* < 0.05—a,c,e,f,g,j,n,4,8, ^*∗∗*^*p* < 0.01—b,d,h,i,k,l,o,1,2,3,5,6,7, ^*∗∗∗*^*p* < 0.001—m. For *average fixation*, ^*∗∗*^*p* < 0.01—8, ^*∗∗∗*^*p* < 0.001—1,2,3,4,5,6,7. For *fixation count*, ^*∗*^*p* < 0.05—f,l,o,t,w,y, ^*∗∗*^*p* < 0.01—a,c,d,e,j,m,n,p,3,7,8,9,10,11, ^*∗∗∗*^*p* < 0.001—b,g,h,i,k,r,s,u,x,z,1,2,4,5,6.

## Data Availability

The data used to support the findings of this study are available from the corresponding author upon request.
